# Repositioning Microtubule Stabilizing Drugs for Brain Disorders

**DOI:** 10.3389/fncel.2018.00226

**Published:** 2018-08-08

**Authors:** Artemis Varidaki, Ye Hong, Eleanor T. Coffey

**Affiliations:** Turku Centre for Biotechnology, Åbo Akademi University and University of Turku, Biocity, Tykistokatu, Turku, Finland

**Keywords:** microtubules, cytoskeleton, kinase, psychiatric disorder, schizophrenia, depression, neurodegenerative disease, cancer

## Abstract

Microtubule stabilizing agents are among the most clinically useful chemotherapeutic drugs. Mostly, they act to stabilize microtubules and inhibit cell division. While not without side effects, new generations of these compounds display improved pharmacokinetic properties and brain penetrance. Neurological disorders are intrinsically associated with microtubule defects, and efforts to reposition microtubule-targeting chemotherapeutic agents for treatment of neurodegenerative and psychiatric illnesses are underway. Here we catalog microtubule regulators that are associated with Alzheimer's and Parkinson's disease, amyotrophic lateral sclerosis, schizophrenia and mood disorders. We outline the classes of microtubule stabilizing agents used for cancer treatment, their brain penetrance properties and neuropathy side effects, and describe efforts to apply these agents for treatment of brain disorders. Finally, we summarize the current state of clinical trials for microtubule stabilizing agents under evaluation for central nervous system disorders.

## Neurons are highly specialized cells that show unique dependencies on microtubules

Neuronal cells are highly compartmentalized consisting of a soma, axon(s), dendrites, and synapses. These compartments develop because of particular cytoskeletal arrangements that are specific to neurons (Witte and Bradke, 2008). Axons convey long distance electrical signals leading to neurotransmitter release from nerve terminal active zones. They vary in length from around 1 mm in hippocampal neurons, to 1 m in certain motor neurons; posing a long distance transport challenge. To overcome this, neurons develop a highly specialized transport system constructed from microtubule polymers and microtubule binding proteins (Matamoros and Baas, 2016; Zahavi et al., 2017). Polymers form from alpha/beta tubulin dimers for which several isoforms exist in brain (Cleveland et al., 1978; Gozes and Littauer, 1978). Microtubules display alternating shrinkage and growth controlled by GTP hydrolysis, a process that is inhibited by binding of microtubule-associated protein (MAPs). Several classes of MAPs exist in the nervous system that confer rigidity to microtubule tracks and enable motor proteins to travel long distances carrying cargo (e.g., mitochondria, proteins, and RNA granules) from soma to dendrites, synapses and axonal terminals. Microtubules themselves undergo post-translational modifications, phosphorylation, acetylation, tyrosination, and polyglutamylation (Marchisella et al., 2016), that mark them for binding by specific proteins, in particular motor proteins (Verhey and Gaertig, 2007). Cargo transport in neurons is directional, kinesin motors transport cargo toward axon terminals (anterograde) and dynein motors carry cargo away from axon tips (retrograde) (Vale, 2003).

Microtubules play fundamental roles in diverse cellular processes, including polarization, migration, cell division, and perhaps most crucially in mature neurons, microtubules facilitate cargo transport. It is therefore not surprising that a large number of genetic variants have been identified that influence healthy functioning of the microtubule cytoskeleton, beyond tubulin isoforms themselves or MAPs. Such microtubule regulators include protein kinases and other post-translational modifying enzymes and signaling proteins, motor proteins, adaptors, ligases, chaperones, scaffolds, and adhesion molecules. Genetic disruption of many of these regulators is associated with pathological disturbance of the neuronal cytoskeleton (summarized in Table 1).

**Table 1:**
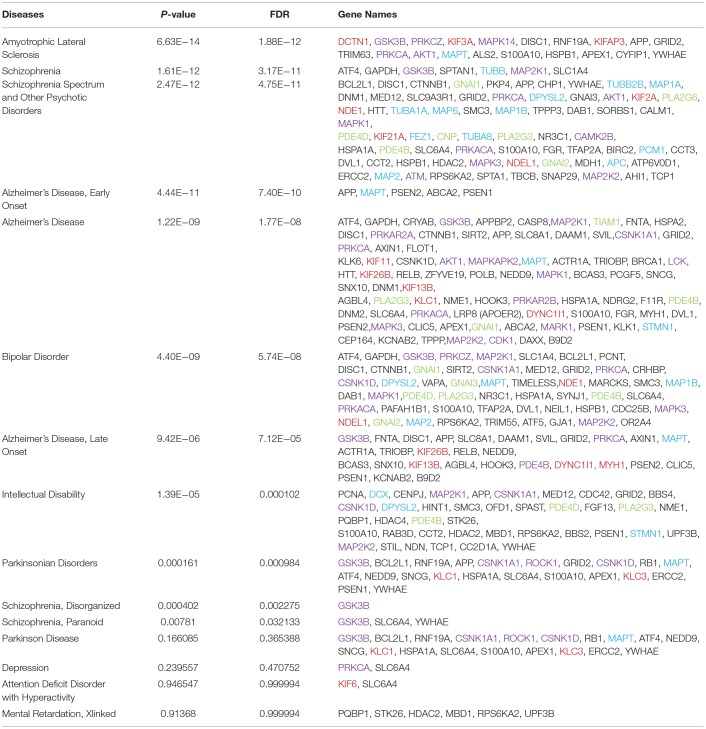
Disease enrichment for “*microtubule regulation and disease*”.

Neurons are particularly susceptible to microtubule defects and deregulation of the microtubule cytoskeleton occurs in a range of neurodegenerative disorders (Matamoros and Baas, 2016). These include Parkinson's and Alzheimer's disease, amyotrophic lateral sclerosis (ALS) and Parkinsonian disorders e.g., progressive supranuclear palsy, all of which have been linked to polymorphisms in the microtubule stabilizing protein *TAU* (*MAPT*) (Zhang et al., 2017). Neurofibrillary lesions consisting of insoluble Tau (MAPT) filaments form in brains from patients with Alzheimer's, Parkinson's, Pick's disease and in Purkinje cell degeneration and ALS (Goedert et al., 2017). Not only neurodegenerative diseases, but also migration disorders can result from microtubule anomalies. Lissencephaly is one such condition which is linked to genetic aberrations in genes encoding tubulin isoforms and tubulin stabilizing proteins, for example doublecortin (Liu, 2011). It results in cortical lamination defects that produce severe intellectual disability and reduced lifespan (Reiner and Sapir, 2013). Crucially also, genetic mutations in tubulins, MAPs, microtubule regulatory proteins and microtubule motor proteins are among the risk genes for schizophrenia spectrum disorders and depression (Marchisella et al., 2016).

## Brain disorders are enriched for defects in genes encoding microtubule regulators

To realize the extent to which microtubule dysregulation is involved in brain disorders, we utilized. Thomson Reuter's MetaCore gene enrichment tool and database that sources disease associations from clinical studies and animal disease models. MetaCore classification of microtubule regulators incorporates a broad range of genes that regulate or associate with microtubules, including non-classical regulators. Two such examples are BRCA1, which regulates γ-tubulin, leading to impaired microtubule nucleation (Sankaran et al., 2007), and KIF26B which lacks the ATPase activity of its motor homologs, but associates with microtubules. Searching for the term “microtubule regulation and disease,” several brain diseases emerged with significant enrichment for genes in this category.

ALS showed the highest enrichment for “microtubule regulation and disease” genes, among brain diseases. ALS-associated genes encoded five protein kinases, three microtubule motors and Tau (MAPT) as well as chaperones and scaffolds. The diseases that showed the next highest level of enrichment were schizophrenia and psychotic disorders. Schizophrenia is a heritable disorder where several gene variants combine to confer disease risk. It is interesting that schizophrenia showed the highest percent of genetic associations for tubulin isoforms and MAPs, among brain diseases, while protein kinases and other signaling proteins were also highly represented. Of particular interest, sixty percent of the “microtubule regulation and disease” genes that associated with bipolar disorder corresponded with schizophrenia spectrum disorders, suggesting that these genes may contribute to overlapping negative symptoms. Finally, the disease that associated with the largest overall number of known “microtubule regulation and disease” genes was Alzheimer's disease, which was associated with 82 genes fitting this search term.

It is worth noting that the genetic landscape of Alzheimer's and Parkinson's disease have not been studied as extensively by GWAS as has the classical polygenic disorder, schizophrenia. Instead, Alzheimer's investigations have focused more on a small number of high penetrance gene mutations that cause autosomal dominant Alzheimer's disease. Yet as this accounts for under 10% of cases, disease-spectrum approaches to study Alzheimer's are relevant, and improved technologies increase feasibility (Van Cauwenberghe et al., 2016). Nonetheless, with current information and straightforward bioinformatics analysis, a higher proportional association of microtubule proteins and MAPs is found for psychiatric disorders (schizophrenia and bipolar, 17 and 7.4% respectively) than for Alzheimer's disease (2.4%). Overall, this enrichment for microtubule regulator genes among major brain disorders emphasizes the importance of proper microtubule regulation for brain health.

## Microtubule stabilizing agents in the clinics

**Figure 1:**
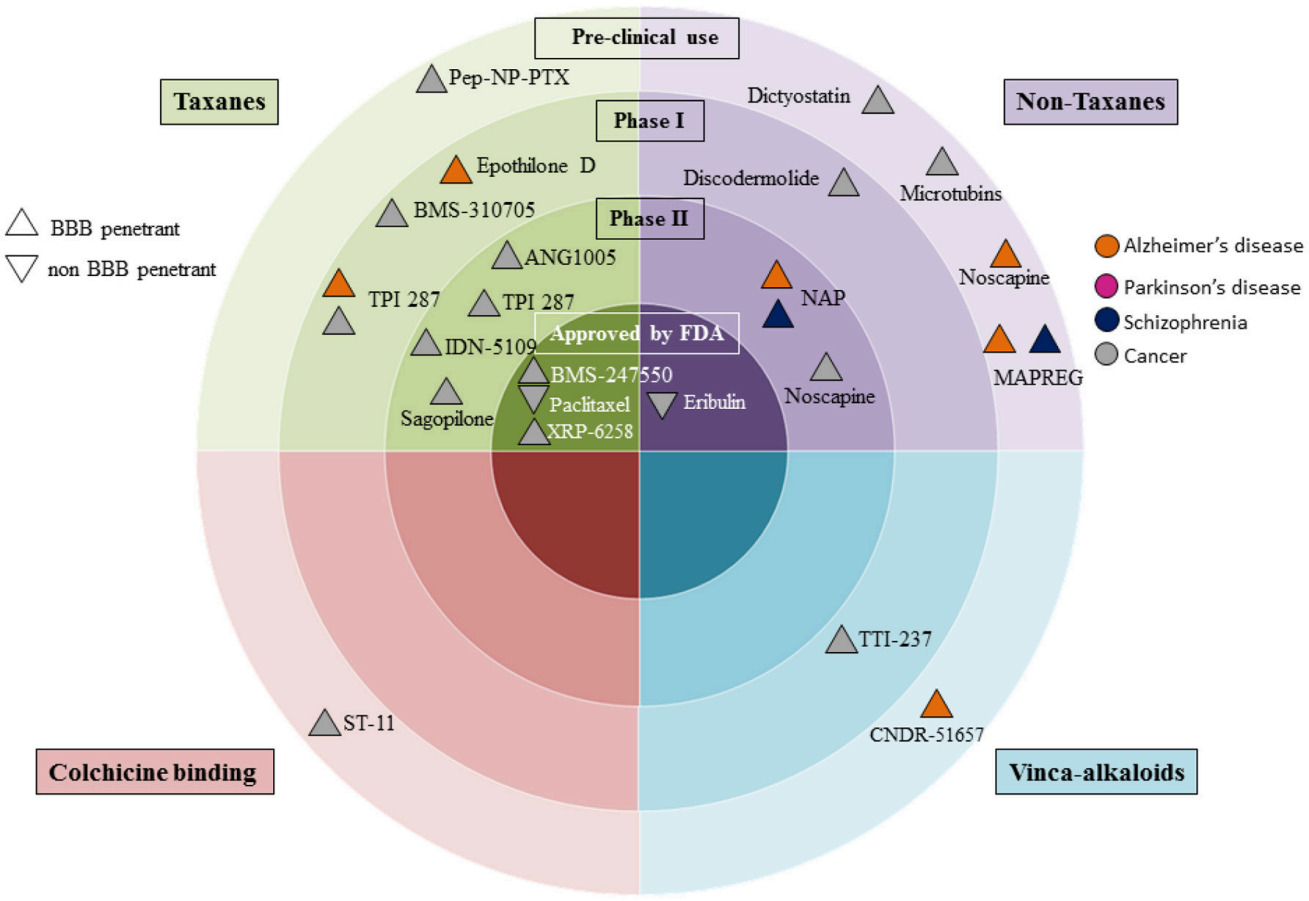
Clinical development of microtubule stabilizing agents for Alzheimer's and Parkinson's disease, schizophrenia and cancer. Microtubule stabilizing agent grouping is according to pre-clinical, or clinical phases. Drugs developed for cancer are in gray; those for Alzheimer's and Parkinson's diseases are coded orange and pink respectively, and those under development for treatment of schizophrenia are in dark purple. We denote blood brain barrier (BBB) penetrance with upright triangles and non-penetrant drugs with an upside down triangle.

The realization that microtubule dysfunction is associated with neuronal disorders, has fuelled efforts to reapply knowledge gained from cancer therapies, where tubulin polymerisation inhibitors have been used for over 50 years. These drugs are under investigation for the treatment of neurodegenerative and psychiatric disorders, as can be seen from the flurry of clinical trial activity in this area (Figure 1; Table 2). The story begins with taxol, one of the earliest recognized microtubule stabilizing agents.

**Table 2:** Clinical trials describing brain penetrance and nervous system side effects for microtubule stabilizing agents.

**Name(s)**	**Cytoskeletal target**	**Diseases**	**Clinicaltrials.gov identifier (Status- study completion date)**	**BBB penetrance/side effects**
Epothilone D (KOS-862, NSC-703147, desoxyepothilone B BMS-241027)	Tubulin	Alzheimer's Disease	NCT01492374 (C) (October 2013)	Brain penetrant/Neurotoxicity severe diarrhea
		Lung cancer	NCT00080509 (T) (November 2004) NCT00081107(C) (December 2004)	
		Colorectal cancer	NCT00077259 (C) (September 2004)	
		Breast cancer	NCT00337649 (C) (April 2008)	
		Prostate cancer	NCT00104130 (T) (February 2005)	
BMS-247550 (ixabepilone)	Tubulin	Breast Cancer	NCT00082433 (C) (March 2008) NCT00789581 (C) (October 2013)	Brain penetrant/CIN, neutropenia, nausea, fatigue, arthralgia, alopecia
BMS-310705	Tubulin	Solid tumors	Phase I (Sessa et al., 2007) (C)	Brain penetrant/neurotoxicity, severe diarrhea
NAP (AL-108, AL-208, davunetide NAPVSIPQ)	EB1/EB3	Schizophrenia	NCT00505765 (C) (April 2009)	Brain penetrant/One patient reported palpitations
		Taupathies	NCT01056965 (A) (July 2017)	
		Mild cognitive impairment in Alzheimer's disease	NCT00422981 (C) (January 2008) NCT00404014 (C) (June 2008)	
		PSP	NCT01110720 (C) (December 2012) NCT01049399 (C) (November 2011)	
TPI 287 (ARC-100)	Unknown	Alzheimer's Disease	NCT01966666 (A) (Est. completion: March 2019)	Brain penetrant/ Seizures, Grade III CIN
		Corticobasal Syndrome, PSP	NCT02133846 (A) (Est. completion: March 2019)	
		Neuroblastoma	NCT00867568 (C) (February 2016) NCT01483820 (T) (December 2014)	
		Metastatic melanoma	NCT01067066 (T) (July, 2016)	
Paclitaxel	Taxol-binding domain	Approved for ovarian, breast and non-small cell lung carcinomas, AIDS-related Kaposi's Sarcoma	Numerous clinical trials	Non-brain penetrant/CIN, neutropenia
				
ANG1005 (GRN1005)	Taxol-binding domain	Brain metastasis	NCT01497665 (T) (February 2013) NCT02048059 (C) (September 2017) NCT01967810 (C) (September 2017) NCT01480583 (C) (October 2015)	Brain penetrant/neutropenia
Sagopilone (ZK-EPO, ZK 219477)	Tubulin	Brain metastasis	NCT00496379 (T) (January 2012	Brain penetrant/fatigue, CIN, nausea,diarrhea, leucopenia hepatobiliary disorder
		Recurrent Glioblastoma	NCT00424060 (C) (August 2007)	
		Metastatic melanoma	NCT00598507 (C) (January 2013)	
Triazolopyrimidines, Cevipabulin	Vinca site	Advanced malignant solid tumors	NCT00195247 (T) (Not mentioned) NCT00195325 (T) (Not mentioned)	Non brain penetrant/Not reported
IDN-5109 (BAY 59-8862, Ortataxel)	Tubulin	Recurrent glioblastoma	NCT01989884 (S) (December 2016	Brain penetrant/Not reported
		Taxane-refractory NSCLC	NCT00054314 (C)(April 2003) NCT00044538 (C) (June 2004)	
		Metastatic breast cancer	NCT00044525 (C) (February 2004)	
		Refractory Non-Hodgkin's Lymphoma	NCT00044551 (C) (July 2003)	
		Renal cell carcinoma	NCT00044564 (C) (January 2003)	
Cabazitaxel (Jevtana, XRP-6258)	Tubulin	Prostate cancer	NCT00417079 (C) (September 2009)	Brain penetrant/ hypotension, bronchospasm, generalized rash/erythema, severe diarrhea, neutropenia, neurotoxicity fatigue, alopecia, Grade 1 neurotoxicity
		Head and neck cancer	NCT01620242 (C) (April 2015)	
		Breast cancer	NCT01934894 (T) (April 2017) NCT03048942 (A) (Est. completion: August 2022)	
		NSCLC	NCT01438307 (C) (September 2015) NCT01852578 (C) (August 2013)	
		Refractory Glioblastoma Multiforme	NCT01866449 (C) (August 2017)	

## Paclitaxel (TAXOL)

Paclitaxel commonly known as “Taxol” is a member of the taxane family. It is a natural compound, antineoplastic drug that was isolated from the bark of the Pacific yew tree (*Taxus brevifolia)* by the botanist Arthur Barclay in 1962, as part of a National Cancer Institute plant-screening program carried out in collaboration with the U.S. Department of Agriculture from 1960 to 1980. Taxol was shown to have cytotoxic activity in 1964 and its structure was resolved in 1971 (Wani et al., 1971). Extensive cancer cell anti-mitotic properties were characterized before taxol entered the clinics, 30 years after its discovery (Kingston, 1991). Nowadays, it is a first-line chemotherapy anti-cancer agent for treatment of breast and ovarian cancers (Wani et al., 1971). It exerts a broad-spectrum anti-tumor activity that is effective in both solid and dissociated tumors. Paclitaxel promotes microtubule assembly *in vitro* (Schiff and Horwitz, 1980) and reduces microtubule dynamics. To do this, it binds a single site on the β-tubulin subunit, on the inner surface near to the lateral protofilament interaction sites (Nogales et al., 1995, 1998). Taxol binding prevents compaction at the tubulin dimer interface and results in more stable lateral interactions. This reduces stochastic switching of microtubules between growth and shrinkage, a classical behavior known as “dynamic instability,” which is essential for normal functioning of the microtubule cytoskeleton (Alushin et al., 2014).

## Blood brain barrier penetrance

One major drawback for the potential use of paclitaxel in the treatment of neurological and neuropsychiatric diseases is the limited bioavailability of this compound in brain. Taxol does not cross the blood brain barrier. Moreover, taxane family compounds are good substrates for P-glycoprotein transporters or multidrug resistance proteins, which are highly expressed in capillary endothelial cells that form the blood brain barrier (Schinkel et al., 1994; Fellner et al., 2002). P-glycoprotein transporters rapidly export taxanes out from cells into the bloodstream, reducing brain bioavailability and contributing to drug resistance. Smart delivery of paclitaxel in the brain has been investigated. For example, taxol conjugation to the brain delivery peptide Angiopep-2 was tested in efforts to improve its blood brain barrier permeability (Régina et al., 2008; Kurzrock et al., 2012). Angiopep-2 is a 19-mer peptide that binds to the lipoprotein related protein-1 receptor and triggers transcytosis. This approach can facilitate targeting of non-blood brain barrier penetrant drugs to the brain, as it provides a mechanism for the conjugated drug to cross the capillary epithelial cell layer. It may also assist selective targeting of cancer cells that exhibit high expression levels of the lipoprotein-related protein receptor (US patent: WO2004060403 A2). ANG1005 (also known as GRN1005; US patent: US7557182 B2), is a conjugate of Angiopep-2 with paclitaxel. It underwent phase II clinical trials to determine its efficacy against breast cancer and non-small cell lung cancer primary tumors, and in patients suffering from high-grade gliomas (NCT 01497665, NCT 02048059, NCT 01967810, and NCT 01480583). In patients with non-small lung cancer with brain metastasis, the prevailing side effect of this drug was peripheral neuropathy, observed in 37.50% of patients, and neutropenia, which affected 18.75% of patients (Table 2).

Another experimental delivery approach is paclitaxel-loaded nanoparticles conjugated to the Pep-1 peptide (Pep-NP-PTX). This delivery system introduced taxol to the brain *via* endocytosis of the interleukin-13 receptor subunit alpha-2 (IL-13RA2), which showed elevated expression in gliomas (Patent application: CN103655517 A). In mice, Pep-NP-PTX showed increased uptake to brain and increased cytostatic effect in glioma cells with no overt toxicity, suggesting that it could represent a viable delivery option (Wang et al., 2015). Yet as discussed below, peripheral neuropathy remains an expected side effect with paclitaxel.

## Chemotherapy-induced peripheral neuropathy

Chemotherapy-induced peripheral neuropathy (CIPN) is one of the most common adverse side effects of paclitaxel treatment. 30–80% of patients will most likely develop CIPN, depending on the dose and duration of the treatment. Most often, these patients will continue to have CIPN symptoms even after discontinuing the treatment (Cavaletti and Marmiroli, 2010). Paclitaxel can induce acute signs of CIPN as early as 24 h after a single high dose treatment. In order to eliminate this side effect, researchers have studied the benefit of using albumin-coated paclitaxel (ABI-007-Abraxane, also known as nab-paclitaxel), a Food and Drug Administration (FDA)-approved medicine for breast, lung and pancreatic cancer (Ibrahim et al., 2005; Nyman et al., 2005). Albumin-bound paclitaxel produced fewer >grade-3 neuropathies, neutropenia and myalgia in non-small cell lung carcinoma patients than did soluble paclitaxel in a phase III trial (Socinski et al., 2012). However meta-analysis of Abraxane use for treatment of breast cancer (including 2357 patients) concluded that the complete response rate was increased but CIPN side effects were aggravated compared to classical taxanes (Zong et al., 2017). Also, in a phase III study of metastatic breast cancer (NCT 00046527), grade-3 sensory neuropathy was increased in patients receiving Abraxane compared to paclitaxel, although this could be attributed to the higher dose of Abraxane used (Gradishar et al., 2005).

Understanding the mechanism whereby paclitaxel induces CIPN may be beneficial in developing strategies to avoid or ameliorate symptoms of neuropathic pain in patients treated with microtubule stabilizing agents. To this end, it was recently shown that microtubule stabilization by paclitaxel decreases axonal transport and downregulates translation of *bclw* mRNA in axons. This in turn leads to activation of a degenerative cascade triggered by mitochondrial dysfunction and activation of the proteolytic enzyme calpain and subsequent axonal degradation (Pease–Raissi et al., 2017). Cognitive deficit is a more recently recognized side effect of chemotherapy, especially in elderly patients (Mandilaras et al., 2013). It will be interesting to see whether drugs with reduced CIPN will also show less cognitive side effects.

## Cabazitaxel

Cabazitaxel (Jevtana, XRP-6258) is a second-generation microtubule-binding drug from the taxane family. It received approval by the FDA in 2010 for the treatment of refractory metastatic prostate cancer (Abidi, 2013). A phase III study (NCT 00417079) of patients with metastatic prostate cancer showed that Cabazitaxel prolonged the survival rate of the patients, with only 1% of patients suffering from grade-3 peripheral neuropathy (de Bono et al., 2010). Cabazitaxel is an attractive anticancer compound compared to paclitaxel as it displays poor affinity for the P-glycoprotein receptor multi-drug resistance pump and as a consequence is blood-brain penetrant (Cisternino et al., 2003). As such, phase II trial (NCT 01866449) used it for treatment of refractory glioblastoma multiforme. Cabazitaxel underwent evaluation for its efficacy in ameliorating pediatric brain tumors such as atypical teratoid rhabdoid tumors, medulloblastomas, and central nervous system primitive neuroectodermal tumors (Girard et al., 2015). Like classical taxanes, cabazitaxel exerts anti-proliferative and pro-apoptotic effects in glioma while disrupting cell cycle by its effects on the microtubule cytoskeleton. Remarkably, it does this at a dose that is non-toxic to surrounding primary neurons and astrocytes (Ghoochani et al., 2016), suggesting that low levels could be viable for treatment of microtubule disorders in brain, in addition to tumors.

## Discodermolide and its analog dictyostatin

Discodermolide is a polyhydroxylated lactone that was isolated from the marine sponge *Discodermia dissolute* (Gunasekera et al., 1990). This compound stabilizes microtubules more potently than paclitaxel and retains anti-tumor activity in cell lines that express high levels of P-glycoprotein and are normally resistant to taxanes (Kowalski et al., 1997). Discodermolide binds with higher affinity to tubulin dimers compared to paclitaxel. X-ray crystallography has shown that discodermolide binds the taxane pocket of β-tubulin, but it does this differently to paclitaxel (Sáez-Calvo et al., 2017). More specifically, while paclitaxel binds to the M-loop of tubulin, discodermolide binds preferentially to the N-terminal H1S2 loop, helping to promote a synergistic effect of these two drugs (Martello et al., 2000; Khrapunovich–Baine et al., 2009). Discodermolide has been in phase I clinical trials for treatment of advanced solid malignancies, where patients did not display any severe neuropathy or neutropenia but did show signs of pulmonary toxicity (Mita et al., 2004). The discodermolide analog dictyostatin has comparable properties to discodermolide in terms of tubulin binding and polymerization (Madiraju et al., 2005). However, dictyostatin shows improved brain bioavailability, 55-times higher than that of discodermolide. Dictyostatin increases levels of acetylated tubulin in brains of mice, indicating that it increases microtubule stability *in vivo*, as expected. Indeed, dictyostatin is a more potent microtubule-stabilizing agent when compared to discodermolide (Brunden et al., 2013). This together with its improved pharmacodynamic properties suggest that dictyostatin could be suitable for treating central nervous system disorders.

## Epothilones

The Epothilones are pharmacological agents comprising 16-membered macrocyclic lactones isolated from the soil dwelling bacteria *Sorangium cellulosum* (Hoefle et al., 1993) (Patent number: DE4138042 A1). Like taxol, they bind and stabilize microtubule polymers, promoting stabilization at sub-micromolar concentrations and inducing cytotoxicity through cell cycle arrest (Bollag et al., 1995; Giannakakou et al., 2000; Nettles et al., 2004). Epothilones have additional advantages that confer increased potency compared to other anti-neoplastic agents. They are water-soluble and amenable to large-scale production through bacterial fermentation. Most importantly however, epothilones are also poor substrates for the P-glycoprotein transport pump, thereby increasing brain bioavailability (Bollag et al., 1995; Aller et al., 2009).

## Epothilone analogs and clinical trials

Since its first identification, several epothilone analogs underwent synthesis resulting in higher anti-tumor efficacy compared to paclitaxel (EPO906) and the original natural compound epothilones A and B (Hoefle et al., 1993). Among these, epothilone B shows the highest potency. Analogs of epothilone B, such as epothilone D (BMS-241027) display a maximum tolerated dose of 25 mg/kg compared to 0.3 mg/kg for epothilone B (Chou et al., 1998). Moreover, the activity of BMS-247550 (also known as ixabepilone), a lactam analog of epothilone B, is twice as high as paclitaxel (Lee et al., 2001). Another epothilone B analog, BMS-310705, was developed to improve compound solubility, however clinical development was discontinued due to associated severe toxic side-effects and because ixabepilone received FDA approval for treatment of breast cancer (Overmoyer et al., 2005; Sessa et al., 2007; Brogdon et al., 2014).

Additionally, sagopilone (ZK-EPO, ZK-Epothilone, ZK 219477) was developed as the first synthetic epothilone designed for cancer treatment (Klar et al., 2006). It shows good brain bioavailability (Hoffmann et al., 2009), and entered phase II trial for breast cancer metastasis (NCT 00496379). However, the trial terminated; side effects of this compound included fatigue, nausea, diarrhea, leucopenia, CIPN and hepatobiliary disorders (Freedman et al., 2011).

## Epothilones in animal models of neurodegeneration and spinal cord injury

In experimental studies, epothilones underwent testing against several brain diseases, including tauopathies and Parkinson's disease (Brunden et al., 2011; Ruschel et al., 2015). More specifically systemic injection of a low dose (0.75 mg/mg body weight) of epothilone B to rats following spinal cord injury promoted axonal recovery (Ruschel et al., 2015). Interestingly, in the same experiments, epotheline B also reduced fibrotic scaring. The microtubule-stabilizing drug at once reduced microtubule polarization in fibroblasts, while promoting microtubule polarization in neurons. By this means, it facilitated axon regrowth into the injured area. This ground breaking study suggested that epotheline B could be useful for clinical treatment of CNS injury. A later study addressed the effect of epotheline B on physiological pruning of axonal branches and synapses. Studying the neuromuscular junction, the authors identified that disassembly of synapses was elicited locally by microtubule disassembly and that a single injection with epothilone B slowed this synapse elimination (Brill et al., 2016). The effect attributed to stabilization of microtubules because EB3 comet density reduced and tubulin content increased. Together these studies provided impetus to harness microtubule-stabilizing drugs such as epothilone B for therapy. More recently, epothilone D was shown to facilitate recovery of hind limb function after spinal cord injury in rats (Sandner et al., 2018), yet in the SOD1^G93A^ mouse model of ALS, epothilone D accelerated disease progression (Clark et al., 2018), indicating that more work is needed in a variety of models.

## Potential benefits of epothilones for tauopathies

Study of paclitaxel binding to microtubules revealed that it displaced Tau from microtubules, suggesting that paclitaxel and tau share a common binding site and microtubule stabilization mechanism (Kar et al., 2003). Tauopathies represent a class of neurodegenerative diseases where there is pathological aggregation of Tau. Hyper-phosphorylated Tau, a pathological hallmark of tauopathies, binds more poorly to microtubules and forms filamentous or amorphous aggregates (Iqbal et al., 2010). Epothilones provide a potential means to recover microtubule stability in the absence of this endogenous stabilizer.

Epothilone D has proven beneficial for amelioration of Tau-related pathologies in experimental animal models, where Tau mis-folding leads to impaired microtubule stability. The widely utilized MAPT (Tau)-PS19 transgenic mice are one such example. These mice harbor the disease-associated P301S mutation that gives rise to “4T” Tau, which harbors four microtubule-binding domains. The expression of Tau in PS19 mice is five-fold higher than endogenous levels, and they develop hallmarks of Alzheimer's disease including neuronal loss in the hippocampus, spreading the neocortex and entorhinal cortex. They also develop neurofibrillary-like inclusions accompanied by micro-gliosis, astrocytosis, although notably they do not develop amyloid plaques (Yoshiyama et al., 2007). Low doses of epothilone D (lower than those used in phase II clinical trials), increased microtubule density in MAPT(Tau)-PS19 mice. The drug also reduced axonal dystrophy and enhanced cognitive performance (Brunden et al., 2010). Furthermore, in neuronal cultures from amyloid precursor protein-mice, epothilone D treatment increased dendritic spine density (Penazzi et al., 2016). Because of such findings, epothilone D underwent clinical trial investigation in patients with mild Alzheimer's disease. In these studies, Tau provided a biomarker readout from cerebrospinal fluid; while cognitive performance and functional magnetic resonance imaging were additional outcome measures (NCT 01492374). This trial ended and further investigation of epothilone D discontinued. No results are posted so far (Tables 1, 2; Figure 1).

In the rTg4510 Alzheimer's mouse model, where P301L is conditionally expressed, stochastic microtubule dynamics decrease. Injection with epothilone D recovered baseline dynamics and improved cognitive function in these mice (Barten et al., 2012). A follow up study showed that epothilone D treatment reversed several other features such as microtubule density, axonal dystrophy, and insoluble Tau. Thus, pathological Tau-related hallmarks reduced without any notable side effects in Tau transgenic mice (Zhang et al., 2012). An important finding from these experiments was that epothilone D, by stabilizing microtubule polymers, restored microtubule dynamics. Counter intuitively perhaps, drug-induced stabilization of microtubules did not immobilize them in a way that perturbed normal physiological regulation. This finding increased expectation that this molecule, or its analogs, could provide a beneficial treatment for Alzheimer's disease. However, following a phase I trial that used epothilone D for Alzheimer's disease discontinued, a new trial commenced and results are awaited (NCT 01966666).

## Potential benefit of epothilones for parkinson's disease

Epothilone D treatment ameliorates hallmarks of Parkinson's disease in animal models. Degeneration of nigrostriatal dopaminergic neurons in the substantia nigra pars compacta is the disease-defining hallmark of Parkinson's disease in patients. In recent years, there is emerging evidence that microtubule instability may contribute to the disease etiology. For example MAPT(Tau)-containing neurofibrillary tangles are observed in both Parkinson's and Alzheimer's patients (Bancher et al., 1987; Joachim et al., 1987). Moreover, MAPT(Tau) variants confer genetic risk for Parkinson's disease (Simón–Sánchez et al., 2009). Furthermore, the environmental toxin rotenone, exposure to which is associated with increased risk to develop Parkinson's disease, destabilizes microtubules leading to cell-specific toxicity of midbrain dopaminergic neurons. Microtubule stabilizing drugs such as taxol rescued viability in these neurons (Ren et al., 2005). Similarly, in the 1-methyl-4-phenyl-1,2,3,6-tetrahydropyridine (MPTP) model of Parkinson's disease, epothilone D treatment prevented the loss of dopaminergic neurons in the substantia nigra pars compacta. These effects were attributed to the rescue of microtubule instability induced by MPTP (Cartelli et al., 2013; Killinger and Moszczynska, 2016).

## Potential benefit of epothilones for psychiatric disorders

The effect of epothilone D was tested in mice lacking the gene for microtubule-associated protein-6 (MAP6)/stable tubule only polypeptide (STOP). These mice are used as a model for psychiatric disorders because they exhibit anatomical and behavioral hallmarks associated with schizophrenia that are reversible by treatment with anti-psychotic drugs. For example they display depressed and anxious behavior, altered serotonergic tone and impaired cognitive function (Fournet et al., 2012; Marchisella et al., 2016), while diffusion tensor imaging of MAP6 knockout mice revealed several neuronal tract deficits (Gimenez et al., 2017). In addition, they show impaired sensorimotor gating in the pre-pulse inhibition paradigm, and hyperactivity in a locomotor test, compared to their wild-type littermates (Volle et al., 2013). Promising results were obtained with epothilone D in these mice, where treatment induced an increase in synapse density and improved synaptic function measured by long-term potentiation (Andrieux et al., 2006). While preliminary, the association of anomalies in genes encoding microtubule-stabilizing proteins with psychiatric disorders (Marchisella et al., 2016), suggests that more widespread testing of epothilone and other microtubule stabilizing drugs in similar models is warranted.

## TPI-287—anti-tumor agent in clinical trials for treatment of neurodegenerative diseases

TPI-287 is a third generation taxane (US patents: US7879904 and US7745650). It is a semisynthetic derivative of abeo-taxane and is widely used in cancer therapy. Like other taxanes, TPI-287 binds to tubulin and promotes microtubule stabilization and as a small molecule, it has the ability to cross the blood brain barrier. Experimentally it was investigated as a drug to treat breast cancer that had metastasised to brain. It effectively reduced tumor size after intravenous administration, however unpublished data indicated that there was loss of body weight as a side effect of treatment (Fitzgerald et al., 2012). TPI-287 is in Phase I clinical trial for treatment of mild to moderate symptoms of Alzheimer's disease (NCT 01966666). In this trial, three arms of patients receive different doses of TPI-287, following which patients are monitored for improvement in cognitive function. In addition, Aβ and Tau biomarker expression is measured from cerebrospinal fluid biopsies. The primary goal is to determine the maximum tolerated dose. TPI-287 is also in phase I (NCT 02133846) for treatment of primary 4-repeat tauopathies (4RT), corticobasal syndrome (CBS; or corticobasal degeneration) and progressive supranuclear palsy. Even though both clinical trials are active, and no side effects were reported so far. A completed phase I trial for treatment of neuroblastoma used higher TPI-287 doses (NCT 00867568 and NCT 01483820). These reported that 16.67% of patients showed serious side effects, such as seizures. Also, phase I clinical trial for metastatic melanoma (NCT 01067066) showed that 24% of the patients exhibited grade-3 peripheral neuropathy (McQuade et al., 2016).

## IDN-5109

IDN-5109 (BAY 59-8862, Ortataxel) also belongs to the third generation of taxanes. It is possible to administer orally and it is more active than paclitaxel, displaying also greater solubility (Polizzi et al., 1999; Nicoletti et al., 2000; Jordan et al., 2002). It is a blood-brain barrier penetrant taxane analog, exhibiting a 15-fold higher brain to plasma ratio (Laccabue et al., 2001). Currently, IDN-5109 is in phase II for recurrent glioblastoma (NCT 01989884), taxane-refractory non-small-lung cell carcinoma (NCT 00054314 and NCT 00044538) and metastatic breast cancer (NCT 00044525), refractory NonHodgkin's lymphoma (NCT 00044551) and renal cell carcinoma (NCT 00044564). Clearly, it will be of interest to establish whether neuropathy side effects diminish with this compound. Testing in models of brain disorders is still awaiting.

## Non-taxane group microtubule stabilizers: NAP and D-SAL

NAP, also known as davunetide, is an intranasal neuropeptide (NAPVSIPQ) derived from the activity-dependent neuroprotective protein (ADNP). The minimal 8 amino acid peptide NAP is neuroprotective (Gozes et al., 2005). ADNP mRNA levels have been shown to be dysregulated in Alzheimer's disease and in schizophrenia (Matsuoka et al., 2008; Dresner et al., 2011; Merenlender–Wagner et al., 2015; Ivashko–Pachima et al., 2017; Sragovich et al., 2017), and mutations in the ADNP gene have been found in patients with autism spectrum disorder (Helsmoortel et al., 2014). Clinical symptoms in patients carrying the ADNP mutation, so called Helsmoortel-Van der Aa syndrome or ADNP syndrome, have been well characterized (Van Dijck et al., 2018). The mechanism of action of NAP is believed to involve microtubules. NAP decorates microtubules in cultured cells, although in a purified system, increasing concentrations of NAP failed to induce tubulin polymerization (Yenjerla et al., 2010). More recently however, NAP was shown to interact with microtubule end-binding proteins EB1 and EB3, leading to increased dendritic spine density (Oz et al., 2014). Furthermore, NAP increases Tau binding to EB1/EB3 as part of its cyto-protective mechanism (Gozes et al., 2017, 2018; Ivashko–Pachima et al., 2017). ADNP also participates in the mammalian SWI/SNF complex, which is a multiprotein chromatin-remodeling complex (Mandel and Gozes, 2007).

## Potential benefit of nap for treatment of psychiatric and neurodegenerative disorders

Interestingly, NAP is brain penetrant when administered either by intranasal delivery (AL-108) (Gozes et al., 2000), intravenously (AL-208) (Leker et al., 2002) or intraperitoneally (Spong et al., 2001). NAP can exert its neuroprotective effects at very low concentrations, improving short-term memory in Apo-E mice, a mouse model for Alzheimer's disease (Bassan et al., 1999). Like the L-isomer, the D-amino acid analog of NAP (D-NAP) is also neuroprotective and D-NAP also improves cognitive deficits in Apo-E mice (Brenneman et al., 2004). In the Thy-α-Syn mouse model of Parkinson's disease, where human α-synuclein expression is under the control of the Thy-1 promoter, NAP treatment reduces both phosphorylated tau and α-synuclein aggregates in the substantia nigra (Fleming et al., 2011; Magen et al., 2014). This decrease in hyper-phosphorylated Tau is also evident in a mouse model of Alzheimer's disease, where NAP enhances axonal transport and improves cognitive performance in the Morris water maze (Matsuoka et al., 2008; Jouroukhin et al., 2013). Additionally, NAP promotes microtubule invasion into neuronal growth cones (Oz et al., 2012). Thus, NAP appears to promote microtubule stability by increasing the affinity of microtubules for Tau binding. Notably, NAP blocks phosphorylation of Tau on Ser-262 (Magen et al., 2014), a residue required for EB1/EB3 interaction (Ramirez–Rios et al., 2016). It is therefore likely that reduction of hyper-phosphorylated Tau by NAP is central for its mechanism (Quraishe et al., 2013; Ivashko–Pachima et al., 2017). Moreover, ADNP itself shows potential as a blood biomarker for Alzheimer's disease (Malishkevich et al., 2016).

Significantly, in the context of psychiatric disorders, NAP reverses the hyperactivity of MAP6/STOP null mice, a mouse model for schizophrenia (Merenlender–Wagner et al., 2010). Moreover, NAP treatment prevents neuronal death often seen with clozapine, a commonly used anti-psychotic drug for treatment of schizophrenia (Merenlender–Wagner et al., 2014). Moreover, NAP reduces depressive-like behavior in mice following social isolation, exemplified by reduced immobility in the forced swim test and reduced anhedonic phenotype (Liu et al., 2017). NAP administration in DISC1 knockout mice, a model for schizophrenia, reduces anxiety levels in the elevated plus maze. On the other hand, combined treatment with the antipsychotic drug risperidone, failed to induce the same anxiolytic response. The authors attributed this to occlusion of the NAP binding site on microtubules by risperidone (Vaisburd et al., 2015). This indicates once more that the mode of action of NAP involves microtubules.

NAP is currently in phase II trial (NCT 00505765) for treatment of schizophrenia where the effect of intranasal NAP (AL-108) administration at two different doses is assessed. This derives from positive results in an earlier study using the UCSD performance based skills assessment (UPSA). While there was no significant improvement in cognitive scoring, the UPSA score showed significant improvement in functional capacity among patients (Javitt et al., 2012). A follow-up, double-blind study demonstrated no effect of a high dose of NAP (davunetide) in elevating N-acetylaspartate (Jarskog et al., 2013), a metabolite that is proposed to be slightly reduced in schizophrenia patients, although this is controversial (Steen et al., 2005). Moreover, NAP is in phase II trial (NCT 01056965) to assess its effects on mild cognitive impairment in Alzheimer's disease. Earlier analysis showed that NAP was well tolerated by Alzheimer's patients with no obvious side effects, and there was some improvement in individual memory tasks but not for composite memory score (Gozes et al., 2009; Morimoto et al., 2013). As an extension of the NAP studies, a shorter 4-amino acid peptide termed “SKIP” which is binds the EB binding site of ADNP is being studied in animal models for potential applications in psychiatric disorders (Amram et al., 2016).

## Triazolopyrimidines

Triazolopyrimidines are non-naturally occurring microtubule stabilizing compounds. Rather than acting like other microtubule stabilizing drugs, to stabilize the lateral edges, they bind in the vinca-site, normally targeted by agents such as vinblastine, acting to stabilize the longitudinal contacts between tubulin subunits (Sáez-Calvo et al., 2017). Triazolopyrimidines display similar properties to other microtubule stabilizing agents in promoting microtubule polymerization. Some of these compounds CNDR-51549 and CNDR51555 (US patent: US20170173016 A1) are capable of crossing the blood-brain barrier and increase the acetylated form of tubulin which reflects the stable pool (Lou et al., 2014; Cornec et al., 2015). Moreover, CNDR-51657 is capable of reducing hyperphosphorylated Tau induced by okadaic acid treatment (Kovalevich et al., 2016). Cevipabulin (TTI-237) (Beyer et al., 2008), another compound from this family which is not brain penetrant, has undergone phase I clinical trials for treatment of advanced malignant solid tumors (NCT 00195247 and NCT 00195325).

## Conclusions

Finding new treatments for neurological disorders has proven to be an extremely challenging task. The realization that microtubule disruption plays a central role in brain disease opens up the possibility to benefit from knowledge gained from existing cancer therapeutics. Starting with the discovery of taxol in a National Cancer Institute-funded screening program in 1962, an array of brain penetrant microtubule stabilizers were developed with the goal of treating gliomas. Now, microtubule-stabilizing agents are under investigation for treatment of a variety of brain disorders. While this endeavor has not been without hurdles, we can expect that the high demand for therapeutic breakthroughs in this area will drive progress toward successful clinical outcome. In a field where receptor drugs dominate, this represents a completely new departure that looks promising.

## Author contributions

AV, YH, and EC contributed to the conception and writing of the manuscript.

### Conflict of interest statement

The authors declare that the research was conducted in the absence of any commercial or financial relationships that could be construed as a potential conflict of interest.
